# Study on the Associations of Individual and Work-Related Factors with Low Back Pain among Manufacturing Workers Based on Logistic Regression and Structural Equation Model

**DOI:** 10.3390/ijerph18041525

**Published:** 2021-02-05

**Authors:** Yidan Dong, Xu Jin, Jingjing Wang, Nazhakaiti Maimaiti, Lihua He, Fujiang Wang, Xianning Jin, Shijuan Wang, Zhongbin Zhang, Mikael Forsman, Liyun Yang

**Affiliations:** 1 Department of Occupational and Environmental Health, School of Public Health, Peking University, Beijing 100191, China; dongyidan@bjmu.edu.cn (Y.D.); xjin96@pku.edu.cn (X.J.); 13011220922@163.com (J.W.); nazakat@bjmu.edu.cn (N.M.); wangfujiang@bjmu.edu.cn (F.W.); 18801238292@163.com (X.J.); wjuan7355@163.com (S.W.); 2 National Center for Occupational Safety and Health, NHC, Beijing 102308, China; zzb_sd@163.com; 3 Institute of Environmental Medicine, Karolinska Institutet, 17177 Stockholm, Sweden; mikael.forsman@ki.se (M.F.); liyun.yang@ki.se (L.Y.); 4 Division of Ergonomics, KTH Royal Institute of Technology, 14157 Huddinge, Sweden

**Keywords:** ergonomics, awkward postures, psychosocial factors, low back pain, manufacturing workers, structural equation model

## Abstract

Work-related musculoskeletal injuries are one of the major occupational health issues of the workers, especially low back pain (LBP). The aim of this study was to survey the prevalence of LBP among manufacturing workers and to identify associations of individual and work-related factors with LBP. A cross-sectional questionnaire study was performed with 1173 participating manufacturing workers. The questionnaire included individual factors, psychosocial and physical exposures, and musculoskeletal discomfort. It was analyzed by logistic regression and structural equation modeling (SEM). The 1-year prevalence of LBP among Chinese manufacturing workers was 33.6%. Logistic regression analysis showed that job tenure, awkward postures, vibration and job demand were positively—while social support and job control were negatively associated with LBP (*p <* 0.05). The SEM results indicated that, as shown in other studies, job types, job tenure, postural load, high job demand, low job control and vibration were directly associated with LBP, but also that job types, high job demand, low social support and vibration may have indirect effects on LBP—mediated by postural load.

## 1. Introduction

Low back pain (LBP) is a worldwide public health problem. Epidemiological studies have shown a high prevalence and incidence of LBP [1,2,3]. In addition, there are serious individual consequences from LBP induced disability, poor quality of life and sickness absence [4]. There are also consequences in terms of economic burden on society and businesses [5]. LBP and neck pain have been ranked as number four in disability-adjusted life lost years worldwide, and there is an increasing trend [6]. Persistent back pain can not only significantly change the physiological and psychological state of individuals, but it can also cause emotional and cognitive abnormalities, reduce work efficiency, increase the error rate (i.e., reduce work quality) and cause accidents at work [7,8,9]. It causes loss of productive time and frequent absence of employees [10,11,12,13]. According to the Health and Safety Executive Board (HSE), about 1.8 million working days were lost due to back pain in the UK in 2016/17 [14]. 

It is generally agreed that LBP is of multifactorial origin and associated with individual characteristics, biomechanical and psychosocial factors [15,16,17]. Individual factors such as age, gender, tenure, anthropometric parameters, personal habits, etc. seem to pose risks for LBP [18,19]. The most commonly reported biomechanical risk factors with at least reasonable evidence for causing LBP include excessive repetition and awkward postures [20,21]. Several recent epidemiological studies have shown that psychosocial factors (e.g., job demand, job control, and social support) may increase the risk of developing LBP [20,22,23]. Results of studies also have provided evidence for the correlation between psychological factors and biomechanical factors [24,25]. Some studies have shown that vibration also plays an important role in the occurrence and development of LBP [26,27]. These risk factors are not independent and may interact with each other, and the complex relationships between these factors are still unclear. Therefore, it is still of high significance to analyze the factors related to the occurrence and development of LBP, in order to improve risk management and LBP prevention in the workplace. 

Univariate or multiple logistic regression is a traditional method to explore e.g., risk factors of LBP. However, it is not effective when there are many intermediate variables and effect modifiers in the model, so it is not possible to provide a comprehensive assessment of the complex interrelationship between risk factors [24]. As a general framework of statistical analysis, the structural equation model (SEM) is widely used in data analysis of social sciences [28]. SEM is a statistical method for establishing, estimating and verifying causality models. Compared with univariate and multiple logistic regression, SEM has several advantages. Multiple independent and dependent variables can be computed simultaneously. Some potential variables that cannot be measured directly can be processed, and measurement errors can be reduced. The pathway and indirect effect of factors can be analyzed, and the pathogenesis of diseases can be explored. Therefore, SEM is increasingly used to analyze the complex interrelationships between risk factors associated with the development of musculoskeletal diseases [18,24,29,30,31,32].

**Figure 1:**
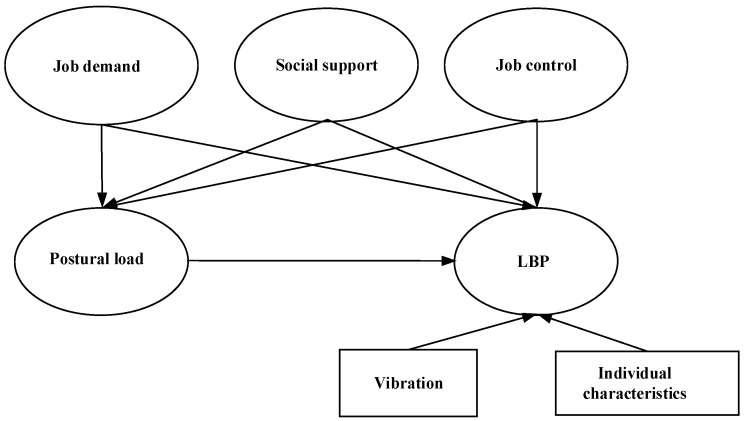
Theoretical framework showing tested influencing factors, low back pain (LBP), and their interrelationships. Note: Rectangles and ovals represent observed and latent variables, respectively.

Based on the theoretical framework describing the assumed interrelationships between individual characteristics, postural load, psychosocial factors and vibration with LBP (Figure 1), an SEM of LBP on sample of manufacturing workers was established and tested. Figure 1 shows that latent variables such as postural load, psychosocial factors and observed variables such as individual characteristics, vibration may have direct effects on LBP, while postural load may be treated as a mediator of the effect of psychosocial factors on LBP. The purpose of this study was to survey the prevalence of LBP among manufacturing workers, and to identify the complex associations of individual and work-related factors with LBP.

## 2. Materials and Methods

### 2.1. Participants

In this study, a cluster sampling method with workshop as the sampling unit was used to select participants from four manufacturing enterprises with unified inclusion-exclusion criteria. The four manufacturing enterprises included three electronic manufacturing enterprises in Beijing and one vehicle manufacturing enterprise in Changchun. The inclusion criteria were (1) at least 18 years old; (2) at least 12 months of employment in the present position; (3) informed consent to participate in this study. The exclusion criteria were (1) workers who reported a history of musculoskeletal injuries in the past; (2) workers who reported rheumatoid arthritis, tumors, tuberculosis, infections, autoimmune diseases and other diseases affecting the musculoskeletal system. All participants meeting eligibility criteria were sent questionnaires, 1222 participants returned completed questionnaires (97.8% response rate). Of the 1222 returned questionnaires, 1173 questionnaires were valid (96.0% efficient rate). 

### 2.2. The Questionnaire

The self-administered Chinese Musculoskeletal Questionnaire (CMQ) was used for evaluating LBP and ergonomic factors in the workplace, which has previously been tested for reliability and validity [33]. It includes three parts: basic personal information, work-related factors and musculoskeletal symptoms, which are described in the following paragraphs. The overall Cronbach’s alpha was 0.808 [33]. 

Basic personal information concerning name, gender, age, job category, job tenure, weight, height, education, monthly income, physical exercise, smoking and drinking behaviors were collected. Body Mass Index (BMI) is calculated as weight in kilograms divided by height in meters squared (kg/m^2^) [34].

Work-related factors including postural factors, psychosocial factors and vibration were assessed. Postural factors were constituted of several items on each body region, which were modified from rapid upper limb assessment (RULA) [35]. Postural items in the low back region included bending your trunk backward frequently (>4 times/min), keeping your trunk in a backward posture for long periods (>1 min), bending your trunk sideways frequently (>4 times/min), keeping your trunk in a side bent posture for long periods (>1 min), bending your trunk frequently (>4 times/min), keeping your trunk in a bent posture for long periods (>1 min), twisting your trunk frequently (>4 times/min), keeping trunk in a twisted posture for long periods (>1 min). Psychosocial factors mainly included job demand, social support and job control, which were selected from the full recommended version of the Karasek Job Content Questionnaire [36]. Job demand included “work fast”, “work hard”, “enough time” and “conflicting demands”. Social support included “coworkers competent”, “coworkers interested in me”, “friendly coworkers”, “coworkers helpful”, “supervisor concerned” and “helpful supervisor”. Job control included “plenty of decision freedom”, “a lot of say”, “develop own abilities” and “allows own decisions”. In addition, the questionnaire also asked participants whether they were exposed to vibration at work. All responses were made on 5-point scales with anchors at 1 (never), 2 (seldom), 3 (sometimes), 4 (often) and 5 (always).

Most of musculoskeletal symptoms items were designed based on the Dutch Musculoskeletal Questionnaire (DMQ) and the Nordic Musculoskeletal Questionnaire (NMQ) [37,38] and have been translated and evaluated in our previous work [39]. Participants were asked if they experienced musculoskeletal symptoms such as ache, pain or discomfort during the past 12 months in a body map with nine body regions: neck, shoulders, upper back, low back, elbows, wrists/hands, hips/thighs, knees and ankles/feet. Furthermore, symptoms in the past 12 months were assessed by self-reported pain duration (never, less than one day, more than one day, more than one week, more than one month), pain frequency (1–2 times/year, 1–2 times/quarter, 1–2 times/month, 1–2 times/week, almost every day), and pain intensity (a 0 to 10 Visual Analogue Scale (VAS): 0 mark as be painless, 1 to 3 marks as mild pain, 4 to 6 marks as moderate pain, 7 to 9 severe pain, and 10 marks as maximum pain) in each body part. 

### 2.3. Data Collection

A cross-sectional descriptive study was conducted in Beijing and Changchun from June to September 2017. Questionnaires accompanied by a cover letter explaining the purposes and procedure of the study were sent to participants. Those who agreed to participate in the study provided their signatures as informed consents. Participants were encouraged to fill in the questionnaires based on their genuine feelings. This study was approved by the local ethics committee of Peking University (Approval identification number: IRB0000105216015). 

### 2.4. Statistical Analysis

#### 2.4.1. Descriptive Statistics and Logistic Regression Analysis

EpiData 3.1 software (The EpiData Association, Odense, Denmark) was used for data entry, and the double-entry method was used to minimize data entry error. Descriptive statistics were performed with SPSS 21.0 (IBM, New York, NY, USA) to describe demographic and occupational characteristics of workers by using count, percentage, and median with interquartile range. The Chi-square test was used to compare the prevalence of LBP in different groups. Binary logistic regression analysis with backward stepwise selection was used to evaluate the associations between LBP and the independent variables (entry criterion: *p* < 0.05, removal criterion: *p* > 0.1). The significance of associations was established at a *p* value of < 0.05 and odd ratios (ORs) with 95% confidence intervals (CI). 

#### 2.4.2. Structural Equation Model

SEM was performed by Mplus 7.0 software (Muthén and Muthén, Los Angeles, CA, USA) to analyze the influence path and effect of the various factors affecting LBP. SEM was established in three stages. First, based on the epidemiological theory model and related risk factors, the initial SEM of LBP was constructed, consisting of the measurement model and the structure model [18,40]. In the hypothesis, latent variables such as postural load and observed variables such as vibration, gender, age, tenure, job types, education, monthly income, physical exercise, smoking and drinking behaviors might affect LBP directly, while latent variables such as job demand, social support, job control might affect LBP directly and indirectly through postural load. 

Next, it was necessary to fit and evaluate the model. The weighted least squares with mean and variance adjusted method (WLSMV) was adopted to estimate the parameters. The measure reliability and convergent validity of the latent variable were evaluated by square multiple correlations (SMC), composite reliability (CR) and the average of variance extracted (AVE). According to previous reports [41], an SMC above 0.5, a CR above 0.7 and an AVE above 0.5 were ideal, and an SMC above 0.36, a CR above 0.6 and an AVE above 0.36 were acceptable. The square root of each construct’s AVE was larger than its correlations with other constructs, which was used to evaluate discriminant validity [42]. The ratio of chi-square to degree of freedom (χ^2^/df), comparative fit index (CFI), Tucker–Lewis index (TLI), root mean square error of approximation (RMSEA) and standardized root mean square residual (SRMR) were used to assess the goodness of the model fit to the data variance/covariance matrix. The fit of the model was considered adequate when χ^2^/df < 3.000, RMSEA < 0.080, SRMR < 0.080, CFI and TLI > 0.900 [43,44].

Finally, when a model was evaluated and fitted poorly, it is necessary to improve and revise the model by referring to professional knowledge and the correction index. A path coefficient γ’s magnitude indicated the strength of the relationship between two latent variables. The direct, indirect and total effect of variables were calculated according to the value of γ in the final model. Statistical significance was set at the 5% level.

## 3. Results

### 3.1. The Demographic Characteristics of Participants 

The 1-year prevalence of LBP among participants was 33.6%. Table 1 reveals the demographic characteristics of participants. It was found that 66.6% participants were males. The median age of participants was 28 years with an interquartile range of 25 to 31 years. Of the study participants, 56.8% worked in their current position for less than 5 years, 59.7% were electronic assemblers, 93.5% were less-educated with an educational level of below college. It was also observed that 87.5% of participants’ monthly income was less than 5000 yuan, 65.6% of participants had exercise habit, 70.4% of participants had smoking behaviors and 81.0% of participants had drinking behaviors. Regarding the BMI of participants, 24.6% were over-weight and 8.2% were obese. The results of chi-square test showed that there were statistically significant differences in the prevalence of LBP among different gender, age, job tenure, job types, BMI, education, monthly income, exercise and smoking groups (*p* < 0.01).

### 3.2. Logistic Regression Model

LBP was defined as positive if participants had lower back symptoms such as pain, discomfort, numbness or limitation of movement during the past 12 months, which lasted for more than 24 h and had no relief after rest. A total of 29 variables of personal and work-related factors were considered into the binary logistic regression model, and 8 variables with statistical significance entered the final model, which are presented in Table 2. Job tenure was found to be positively associated with LBP. The risk of LBP in participants who had worked for 6–10 years was higher than those who had worked for 1–5 years (OR = 1.842, 95% CI = 1.127–3.010). The risk of LBP in participants who had worked for 16 years or more was higher than those who had worked for 1–5 years (OR = 3.404, 95% CI = 1.374–8.434).

Several postural factors were recognized to be positively associated with LBP. The risk of LBP in participants who often bent their trunk sideways frequently was higher than those who never bent their trunk sideways (OR = 2.625, 95% CI = 1.130–6.098). The risk of LBP in participants who seldom bent their trunk frequently was higher than those who never bent their trunk (OR = 2.705, 95% CI = 1.427–5.127). The risk of LBP in participants who always kept their trunk in a bent posture for long periods (OR = 6.442, 95% CI = 1.010–41.082) or sometimes kept their trunk in a bent posture for long periods (OR = 3.328, 95% CI = 1.498–7.395) was higher than those who never kept their trunk in a bent posture. 

Psychosocial factors involving job demand, social support and job control were observed to be associated with LBP. Participants who always worked fast had a higher risk than those never worked fast (OR = 4.760, 95% CI = 1.452–15.603). Participants whose coworkers were sometimes helpful had a higher risk than those whose coworkers were always helpful (OR = 1.884, 95% CI = 1.093–3.249). Participants who always developed their abilities had a lower risk than those never developed their abilities (OR = 0.287, 95% CI = 0.110–0.748). 

In addition, vibration was also found to be positively associated with LBP. Compared with participants who had never been exposed to vibration at work, those who were always exposed to vibration had a higher risk (OR = 2.930, 95% CI = 1.405–6.109).

### 3.3. Structural Equation Model

The measurement model consisted of five latent variables: postural load, job demand, social support, job control and LBP. The reliability and validity of the measurement model are evaluated in Table 3. The item reliability was higher than 0.5, and the composite reliability was higher than 0.7, which showed that all latent variables had good reliability. The convergence validity was higher than 0.5, which indicated that all latent variables had ideal convergence validity. The square roots of all AVEs were above 0.7, which were much larger than all the cross-correlations, indicating that all latent variables had adequate discriminant validity.

**Figure 2:**
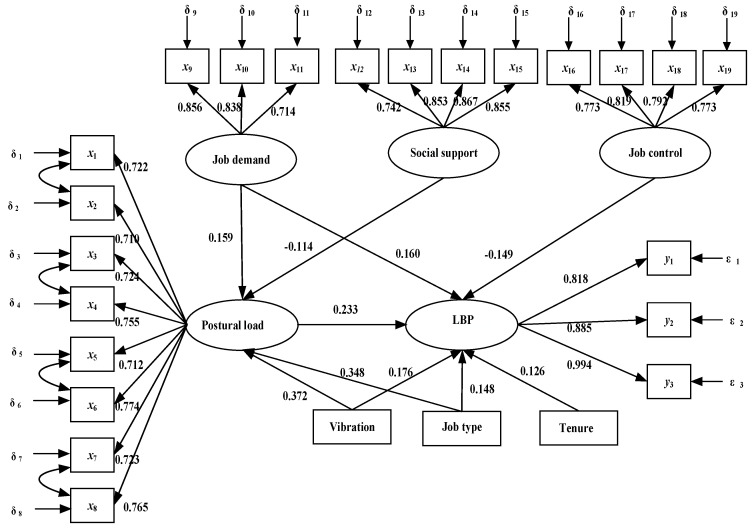
The path diagram of structural equation model. Note: Rectangles and ovals represent observed and latent variables, respectively. LBP represents low back pain.

Figure 2 illustrates the path coefficients of the final SEM model. Results showed that job demand (γ = 0.159, *p* < 0.001), vibration (γ = 0.372, *p* < 0.001) and job types (γ = 0.348, *p* < 0.001) were positively associated with postural load, and that social support (γ = −0.114, *p* = 0.001) was negatively associated with postural load. Job demand (γ =0.160, *p* < 0.001), postural load (γ =0.233, *p* < 0.001), vibration (γ = 0.176, *p* < 0.001), tenure (γ =0.126, *p* = 0.004), job types (γ = 0.148, *p* = 0.004) were positively associated with LBP. Job control (γ = −0.149, *p* <0.001) was negatively associated with LBP. 

The direct, indirect and total effect of variables are shown in Table 4. Job demand had a direct effect (0.160, *p* <0.001) on LBP and had an indirect effect (0.037, *p* =0.001) on LBP through postural load. Social support had an indirect effect (−0.027, *p* = 0.005) on LBP through postural load and job control had a direct effect (−0.149, *p* < 0.001) on LBP. Postural load (0.233, *p* < 0.001), tenure (0.126, *p* = 0.004), vibration (0.176, *p* < 0.001) and job types (0.148, *p* = 0.004) had direct effects on LBP, and vibration (0.087, *p* < 0.001) and job types (0.081, *p* < 0.001) had indirect effects on LBP through postural load. The total effects of tenure, job types, vibration, postural load, job demand and job control on LBP were 0.126, 0.299, 0.262, 0.233, 0.197, and −0.149 respectively. 

Table 5 shows the goodness-of-fit indices. In this material, the ratio of χ^2^/df was calculated to be 2.955. This ratio is an indicator of goodness of fit between the observed and the increased covariance matrices. CFI, which assumes that all latent variables are uncorrelated and compares the sample covariance matrix with the null hypothesis, was 0.944. In addition, the TLI, which is a kind of relative fit index, was calculated to be 0.937. The RMSEA includes the mean of the covariance and variance that was not explained by the model, and in this material, the RMSEA value was found to be 0.048, which indicates an acceptable goodness of fit. The SRMR is the square root of the difference between the residuals of the sample covariance matrix and the hypothesised covariance model, and in this material, the SRMR value was calculated to be 0.075, which also indicates an acceptable goodness of fit.

## 4. Discussion

In this study, we surveyed the prevalence of LBP among Chinese manufacturing workers and identify complex associations of individual and work-related factors with LBP. The 1-year prevalence of LBP in our study was 33.6%, which was comparable to rates reported in the same industry in other studies ranging from 20–60.2% [45,46,47,48]. This may be explained by measurement variance, cultural differences and differences in the perception of terminology may exist among different studies. Compared with other professions, the prevalence of LBP among manufacturing workers was relatively low (in comparison to e.g., nurses and drivers) and relatively high (in comparison to e.g., dentists and teachers), which may partly depend on differences in work posture and workplace, revealing that it is necessary to identify specific hazards in different occupations [49,50,51,52]. 

Our study showed that job types was not only directly related to LBP, but also indirectly related to LBP through postural load. The findings of the study may imply that the difference of postural load may be the main reason for the difference of the prevalence of LBP among different types of workers. In addition, the results of the logistic regression model and SEM showed that job tenure and vibration were positively associated with LBP, which was consistent with previous studies [26,27,53]. A plausible hypothesis is that the higher risk of LBP in workers with longer employment length is a consequence of a longer time exposed to occupational risk factors compared to those who have less employment length [54]. Workers exposed to vibration had a higher risk of LBP may be because the vibration transmitted to the muscles may cause tonic reflex of the muscles and may influence their motor unit synchronization which may affect the muscle internal loads and their fatigue and injury tolerances [55]. Further, SEM provided a new finding that vibration had an indirect effect on LBP through posture load. This may be explained by the assumption that the numbness of the hands/fingers caused by vibration may make it difficult to apply only enough force to control the tools, which may enable workers to better control the tools by changing their work posture [56]. 

The link between awkward postures and LBP has been suggested in many studies [57,58,59,60]. Our findings provided new support to the direct link between awkward postures and LBP. Poor posture may exert a large mechanical load on the low back. Previous research indicated that the intervertebral disc compressive force increases with trunk flexion [61,62,63]. In addition, uneven pressure on the intervertebral discs has been reported when the lumbar vertebrae is in a flexed or an excessively extended position [64]. Thus, poor posture may lead to LBP from a mechanical point of view.

Our study showed a positive association between job demand and LBP, which was in agreement with other studies [20,65,66]. A possible explanation for this result may be that high job demand increase strain, thereby increasing muscle tone, which may lead to musculoskeletal symptoms such as LBP in the long term [40]. In addition, low job control was directly associated with LBP in our study, which was in accordance with earlier studies [66,67,68]. Job control included aspects of skill discretion and decision authority. Skill discretion focused on exerting one’s abilities, decision autonomy concentrated on influential opinions and the ability to independently plan and organize one’s work [36]. Workers with high job control can likely switch to less demanding tasks when they feel overtaxed. However, workers with low job control have no opportunity to escape from demanding tasks and have to continue to exert a high level of effort [69]. As a result, they are more likely to develop LBP than workers with high job control. On the other hand, workers with higher job control may have more autonomy and thus take more short pauses, which can relieve their muscles and reduce the risk of LBP [66]. 

Interestingly, a new finding in our study pointed out that job demand indirectly affects LBP through posture load. A possible interpretation may be that high job demand such as maximum workload and short deadlines was likely to increase hurried movements with high accelerations or poor posture. Additionally, some studies have found support for the association of low social support with LBP [70,71,72]. In contrast, our study indicated no direct relation between social support and LBP, which was similar to the studies from the Sterud and Hartvigsen [66,73]. Our study provided an intriguing finding that social support was indirectly, negatively, associated with LBP through posture load. An explication for the result may be that workers whom receive low social support from managers and co-workers at work bear a larger workload and are more likely to develop LBP. This result needs to be confirmed in further population studies.

The SEM established in this study is not only a further verification of the results of logistic regression analysis, but also shed light on the interrelationship between individual, work-related factors and LBP among manufacturing workers, which may provide a useful information for future studies on the occurrence, causation and prevention of LBP. However, few limitations have not been ruled out in the present study. As this is an exploratory study, the results show only associations. The cross-sectional design limits causal interpretation of the results, and further confirmatory research is needed. In addition, the study sample is limited to specific working segments alone (i.e., electronic manufacturing industry and vehicle manufacturing industry) and may not represent other workforces and industry sectors. Therefore, it is quite advisable to include other industries.

## 5. Conclusions

The prevalence of LBP among Chinese manufacturing workers was 33.6%, which needs our attention. Logistic regression analysis showed that job tenure, awkward postures, vibration and job demand were significantly positively—while social support and job control were negatively associated with LBP. The constructed SEM provided a deeper explanation for the relationship between work-related factors and LBP. The SEM results indicated that, as shown in other studies, job types, job tenure, postural load, high job demand, low job control and vibration were directly associated with LBP, but also that job types, high job demand, low social support and vibration may have indirect effects on LBP—mediated by postural load.

## Figures and Tables

**Table 1:** The demographic characteristics of study participants (N = 1173).

Variables	Categories	Number of LBP Cases (%)	Χ^2^	*p* Value
Gender ^a^	Male	297/748 (39.7)	43.785	<0.001 ***
	Female	75/375 (20.0)		
Age (years old) ^a^	≤20	15/93 (16.1)	30.900	<0.001 ***
	21–30	244/747 (32.7)		
	31–40	88/245 (35.9)		
	≥41	44/79 (55.7)		
Tenure (years) ^a^	1–5	112/610 (18.4)	171.980	<0.001 ***
	6–10	196/349 (56.2)		
	11–15	29/57 (50.9)		
	≥16	37/57 (64.9)		
Job types ^a^	Electronic assembler	114/700 (16.3)	241.698	<0.001 ***
	Vehicle assembler	73/147 (49.7)		
	Riveter and welder	207/326 (63.5)		
BMI (kg/m^2^) ^a^	<18.5	31/121 (25.6)	14.034	0.003 **
	18.5–23.9	206/656 (31.4)		
	24–27.9	104/285 (36.5)		
	≥28	45/95 (47.4)		
Education ^a^	Junior middle school or below	36/147 (24.5)	44.802	<0.001 ***
	Senior high school	161/588 (27.4)		
	Junior college	148/308 (48.1)		
	Bachelor degree or above	26/73 (35.6)		
Monthly income (RMB) ^a^	≤2000	4/12 (33.3)	50.648	<0.001 ***
	2001–4000	172/672 (25.6)		
	4001–5000	124/254 (48.8)		
	≥5001	57/134 (42.5)		
Exercise ^a^	Never	153/379 (40.4)	13.668	0.008 **
	1–3 times/quarter	49/158 (31.0)		
	2–3 times/month	64/216 (29.6)		
	1–2 times/week	67/242 (27.7)		
	More than 3 times/week	35/108 (32.4)		
Smoking ^a^	Yes	243/820 (29.6)	19.161	<0.001 ***
	No	148/345 (42.9)		
Drinking ^a^	Yes	306/940 (49.6)	2.141	0.143
	No	83/220 (48.5)		

Note: BMI = body mass index; LBP = low back pain; ^a^ Variables with missing values; ** *p* < 0.01, *** *p* < 0.001.

**Table 2:** The results of evaluating influencing factors of LBP by logistic regression model.

Variables	Categories	B	S.E.	Wald	*p* Value	OR (95% CI)
Job tenure	1–5 years			10.576	0.014 *	
	6–10 years	0.611	0.251	5.943	0.015 *	1.842 (1.127–3.010)
	11–15 years	0.691	0.437	2.500	0.114	1.995 (0.848–4.696)
	≥16 years	1.225	0.463	7.001	0.008 **	3.404 (1.374–8.434)
Postural load						
Bending your trunk sideways frequently (>4 times/min)	never			8.346	0.080	
	seldom	−0.165	0.263	0.396	0.529	0.848 (0.507–1.418)
	sometimes	−0.260	0.309	0.709	0.400	0.771 (0.421–1.413)
	often	0.965	0.430	5.040	0.025 *	2.625 (1.130–6.098)
	always	−0.210	0.530	0.156	0.693	0.811 (0.287–2.292)
Bending your trunk frequently (>4 times/min)	never			14.291	0.006 **	
	seldom	0.995	0.326	9.299	0.002 **	2.705 (1.427–5.127)
	sometimes	0.127	0.448	0.081	0.777	1.135 (0.472–2.730)
	often	0.931	0.542	2.951	0.086	2.537 (0.877–7.338)
	always	0.729	0.939	0.602	0.438	2.072 (0.329–13.051)
Keeping your trunk in a bent posture for long periods (>1 min)	never			14.693	0.005 **	
	seldom	0.096	0.345	0.077	0.781	1.101 (0.559–2.166)
	sometimes	1.202	0.407	8.710	0.003 **	3.328 (1.498–7.395)
	often	0.914	0.538	2.885	0.089	2.495 (0.869–7.165)
	always	1.863	0.945	3.883	0.049 *	6.442 (1.010–41.082)
Job demand						
Work fast	never			11.371	0.023 *	
	seldom	0.776	0.586	1.755	0.185	2.173 (0.689–6.853)
	sometimes	0.768	0.589	1.702	0.192	2.155 (0.680–6.830)
	often	1.132	0.581	3.798	0.051	3.103 (0.993–9.691)
	always	1.560	0.606	6.633	0.010 **	4.760 (1.452–15.603)
Social support						
Coworkers helpful	always			11.897	0.018 *	
	never	−0.691	0.914	0.572	0.450	0.501 (0.084–3.006)
	seldom	0.475	0.359	1.748	0.186	1.607 (0.795–3.248)
	sometimes	0.634	0.278	5.192	0.023 *	1.884 (1.093–3.249)
	often	−0.205	0.256	0.645	0.422	0.814 (0.493–1.344)
Job control						
Develop own abilities	never			11.548	0.021 *	
	seldom	0.273	0.296	0.851	0.356	1.314 (0.736–2.345)
	sometimes	−0.114	0.312	0.134	0.714	0.892 (0.484–1.644)
	often	0.113	0.388	0.084	0.772	1.119 (0.523–2.394)
	always	−1.247	0.488	6.522	0.011 *	0.287 (0.110–0.748)
Vibration	never			17.168	0.002 **	
	seldom	−0.440	0.310	2.022	0.155	0.644 (0.351–1.181)
	sometimes	−0.045	0.362	0.015	0.902	0.956 (0.471–1.944)
	often	0.553	0.370	2.233	0.135	1.738 (0.842–3.589)
	always	1.075	0.375	8.222	0.004 **	2.930 (1.405–6.109)

Note: * *p* < 0.05, ** *p* < 0.01.

**Table 3:** The reliability and validity of measurement model.

Latent Variable	Item	Item Loading	*p* Value	Item Reliability (SMC ^a^)	Composite Reliability (CR ^b^)	Convergent Validity (AVE ^c^)	Discriminate Validity				
							Postural Load	Job Demand	Social Support	Job Control	LBP
Postural load	x1: Bending your trunk backward frequently (>4 times/min)	0.722	<0.001	0.521	0.904	0.542	**0.736**				
	x2: Keeping your trunk in a backward posture for long periods (>1 min)	0.710	<0.001	0.504							
	x3: Bending your trunk sideways frequently (>4 times/min)	0.724	<0.001	0.524							
	x4: Keeping your trunk in a side bent posture for long periods (>1 min)	0.755	<0.001	0.570							
	x5: Bending your trunk frequently (>4 times/min)	0.712	<0.001	0.507							
	x6: Keeping your trunk in a bent posture for long periods (>1 min)	0.774	<0.001	0.599							
	x7: Twisting your trunk frequently (>4 times/min)	0.723	<0.001	0.523							
	x8: Keeping trunk in a twisted posture for long periods (>1 min)	0.765	<0.001	0.585							
Job demand	x9: Work fast	0.856	<0.001	0.733	0.873	0.633	0.147	**0.796**			
	x10: Work hard	0.838	<0.001	0.702							
	x11: Conflicting demands	0.714	<0.001	0.510							
Social support	x12: Coworkers competent	0.742	<0.001	0.551	0.910	0.669	−0.089	0.128	**0.818**		
	x13: Coworkers interested in me	0.853	<0.001	0.728							
	x14: Friendly coworkers	0.867	<0.001	0.752							
	x15: Coworkers helpful	0.855	<0.001	0.731							
Job control	x16: Plenty of decision freedom	0.773	<0.001	0.598	0.889	0.616	0.004	0.139	0.296	**0.785**	
	x17: A lot of say	0.819	<0.001	0.671							
	x18: Develop own abilities	0.792	<0.001	0.627							
	x19: Allows own decisions	0.773	<0.001	0.598							
LBP	y1: pain duration	0.818	<0.001	0.669	0.925	0.756	0.487	0.171	−0.061	−0.131	**0.869**
	y2: pain frequency	0.885	<0.001	0.783							
	y3: pain intensity	0.994	<0.001	0.988							

Note: SMC = square multiple correlations; CR = composite reliability; AVE = the average of variance extracted; ^a^ SMC >0.5 was ideal, >0.36 was acceptable. ^b^ CR >0.7 was ideal, >0.6 was acceptable. ^c^ AVE >0.5 was ideal, >0.36 was acceptable. The diagonal elements (in bold) represent the square root of the AVE, and the lower triangle is the Pearson correlation of latent variable.

**Table 4:** The direct, indirect and total effect of variables.

Factors	Postural Load			LBP		
	Direct	Indirect	Total	Direct	Indirect	Total
Tenure	—	—	—	0.126 **	—	0.126 **
Job types	0.348 ***	—	0.348 ***	0.148 **	0.081 ***	0.299 ***
Vibration	0.372 ***	—	0.372 ***	0.176 ***	0.087 ***	0.262 ***
Postural load	—	—	—	0.233 ***	—	0.233 **
Job demand	0.159 ***	—	0.159 ***	0.160 ***	0.037 **	0.197 ***
Social support	−0.114 **	—	−0.114 **	−0.016	−0.027 **	−0.043
Job control	—	—	—	−0.149 ***	—	−0.149 **

Note: ** *p* < 0.01, *** *p* < 0.001.

**Table 5:** The model fit test of structural equation model.

Index	Criterion	Research Model
χ^2^/df	χ^2^/df < 3	2.955
CFI	>0.9	0.944
TLI	>0.9	0.937
RMSEA	<0.08	0.048
SRMR	<0.08	0.075

## Data Availability

The data presented in this study are available on request from the corresponding author. The data are not publicly available due to privacy.
